# Echocardiography protocol and cardiometabolic phenotyping in Indian birth cohorts—the IndEcho study

**DOI:** 10.3389/fcvm.2023.1055454

**Published:** 2023-07-13

**Authors:** Senthil K. Vasan, Anoop George Alex, Ambuj Roy, Mahasampath Gowri, Sikha Sinha, Jenifer Suresh, Reesa Susan Philip, Jolly Kochumon, Neetu Jaiswal, Geethanjali Arulappan, Lakshmy Ramakrishnan, Harshpal Singh Sachdev, Nikhil Tandon, Nihal Thomas, Felix Jebasingh, Clive Osmond, Fredrik Karpe, Santosh K. Bhargava, Belavendra Antonisamy, Dorairaj Prabhakaran, Caroline H.D. Fall, Viji S. Thomson

**Affiliations:** ^1^ MRC Lifecourse Epidemiology Centre, University of Southampton, Southampton, UK; ^2^ Oxford Centre for Diabetes, Endocrinology and Metabolism, Radcliffe Department of Medicine, University of Oxford, Oxford, UK; ^3^ Department of Cardiology, Christian Medical College, Vellore, India; ^4^ Department of Cardiology, All India Institute of Medical Sciences, New Delhi, India; ^5^ Centre for Chronic Disease Control, Gurgaon, India; ^6^ Department of Biostatistics, Christian Medical College, Vellore, India; ^7^ Department of Pediatrics, Sitaram Bhartia Institute of Science and Research Institute, New Delhi, India; ^8^ Department of Biochemistry, Christian Medical College, Vellore, India; ^9^ Department of Cardiac Biochemistry, All India Institute of Medical Sciences, New Delhi, India; ^10^ Department of Endocrinology, All India Institute of Medical Sciences, New Delhi, India; ^11^ Department of Endocrinology, Christian Medical College, Vellore, India; ^12^ Department of Pediatrics, Sunder Lal Jain Hospital, New Delhi, India; ^13^ Public Health Foundation of India, New Delhi, India

**Keywords:** echocardiography, Asian Indian, cardiac size, systolic function, diastolic function

## Abstract

**Background:**

Asian Indians are at higher risk of cardiometabolic disease compared to other ethnic groups, and the age of onset is typically younger. Cardiac structure and function are poorly characterized in this ethnic group. In this study, we describe image-acquisition methods and the reproducibility of measurements and detailed echocardiography characteristics in two large Indian population-based cohorts (the New Delhi and Vellore Birth Cohorts) from India.

**Methods:**

The IndEcho study captured transthoracic echocardiographic measurements of cardiac structure and function from 2,322 men and women aged 43–50 years. M-mode measurements in the parasternal long axis (PLAX) and 2-dimensional (2D) short axis recordings at the mitral valve, mid-papillary and apical level were recorded. Apical 2D recordings of two- three- and four-chamber (2C, 3C and 4C) views and Doppler images (colour, pulsed and continuous) were recorded in cine-loop format. Left ventricular (LV) mass, LV hypertrophy, and indices of LV systolic and diastolic function were derived.

**Results:**

Echocardiographic measurements showed good/excellent technical reproducibility. Hetero-geneity across sites, sex and rural/urban differences in cardiac structure and function were observed. Overall, this cohort of South Asian Indians had smaller LV mass and normal systolic and diastolic function when compared with published data on other Asian Indians and the West, (LV mass indexed for body surface area: Delhi men: 68 g/m^2^, women 63.9; Vellore men: 65.8, women 61.6) but were within ethnic-specific reference ranges. The higher prevalence of obesity, diabetes and hypertension is reflected by the higher proportion of LV remodelling and lesser hypertrophy.

**Conclusions:**

Our study adds to scarce population-based echocardiographic data for mid-life Asian Indians. Compared to published literature on other ethnic groups, the Asian Indian heart is characterised by smaller cardiac dimensions and normal range systolic and diastolic function on a background of a high prevalence of hypertension, diabetes and cardiac disease at a relatively young age. This data will form the basis for further analyses of lifecourse, metabolic and body composition predictors of cardiac structure and function, and echocardiographic predictors of future mortality.

**ISRCTN registration number:**

13432279

## Introduction

Cardiovascular disease (CVD) is a major cause of premature mortality globally, accounting for about 52% in India and less than 20% in high income countries (1–3). Differences in CVD mortality and morbidity exist between various ethnic groups (4, 5). South Asian Indians develop CVD at a younger age and lower body mass index (BMI) compared to Europeans/white caucasians (1). These differences have been attributed to numerous factors, including the coexistence of and interaction with key drivers of CVD such as lifestyle factors, diabetes, hypertension and obesity (3). Left ventricular (LV) geometry and function are associated with CVD and mortality independent of conventional risk factors (6) but there is sparse data on LV structure and function, and their relationship to these risk factors, in Asian Indians.

The IndEcho study was designed to evaluate the associations of birth size, early childhood growth and adult CVD risk factors with mid-life cardiac structure and function in South Asian Indians, assessed using transthoracic echocardiography (7). In the current paper, we describe the image-acquisition methodology in over 2,000 participants aged 43–50 years with the aims of providing (1) descriptive data of cardiac structure and function in the cohort, (2) descriptive data of these measures in an apparently healthy sub-set free of CVD, diabetes and hypertension and (3) reproducibility data for the measurements.

## Materials and methods

The IndEcho study used standardized protocols, in accordance with the 2015 Joint Update of American Society of Echocardiography (ASE) and European Association of Cardiovascular Imaging (EACVI) (8), to measure left ventricular (LV) and left atrial dimensions, systolic and diastolic function. Measurements were recorded by trained and experienced sonographers (two each in Delhi and Vellore) under the regular supervision of cardiologists, using transthoracic echocardiography on a Philips CX50 Compact Xtreme system equipped with an IPx-7, S5-1 (Cardiac Sector Probe) transducer. M-mode measurements in the parasternal long axis (PLAX) and short axis recordings at mitral valve, mid-papillary and apical levels were recorded. Apical 2D recordings of two- four- and three chamber (2C, 4C and 3C) views and Doppler images (colour, pulsed and continuous wave on three consecutive cardiac cycles) were recorded in cine-loop format. After joint training sessions before the study started, the measurements were recorded at the two centres independently, but all images were analysed using a Freeland digitizer and software packages (Alpharetta, USA) by a single echo-technician in Vellore (JS). A list of all cardiac parameters measured are provided in Table 1.

**Table 1:** IndEcho echocardiographic measurements.

	Primary measures	Derived measures
Cardiac size	LV wall thickness	Relative wall thickness (RWT) = [(2 X PWT)/LVIDD])
	LVIDD, LVISD	Fractional shortening = (LVIDd-LVIDs)/LVIDd
	Septal thickness	
	Relative wall thickness	
	LVEDV, LVESV	
	LV mass	
Systolic function	Global longitudinal strain	LV-ejection fraction = (LVEDV-LVESV)/LVEDV
Diastolic function	E	E/A ratio = E wave/A wave
	A	Average E/e’ = Average medial E/average lateral e'
	Medial e’	LA volume - LA volume indexed to BSA
	Lateral e’	
	TR max	
Definition of categories		
LV mass category		
Concentric re-modelling	LV mass ≤102 g in men or ≤88 g in women AND RWT > 0.42	
Concentric hypertrophy	LV mass >102 g in men or >88 g in women AND RWT > 0.42	
Eccentric hypertrophy	LV mass >102 g in men or >88 g in women AND RWT ≤ 0.42	
No remodeling or hypertrophy	LV mass ≤102 g in men or ≤88 g in women AND RWT ≤ 0.42	
GLS categories		
Normal GLS	<minus 17%	
Borderline GLS	minus 17–minus 15	
Abnormal GLS	>minus 15%	
LV diastolic dysfunction		
None		
Indeterminate	if exactly two of the following abnormalities are present: 1) septal (medial) e' <7 cm/sec or lateral e' <10 cm/sec; 2) E/e' >14; 3) LA volume >34 ml/m2; and 4) TRmax >2.8 m/sec.	
Dysfunction	if more than two of the following four abnormalities were present: 1) septal (medial) e' <7 cm/sec or lateral e' <10 cm/sec; 2) E/e' >14; 3) LA volume >34 ml/m2; and 4) TRmax >2.8 m/sec.	

LV, left ventricle; PWT, posterior wall thickness; LVIDd and LVIDs, left ventricular internal diameter in diastole and systole; LVEDV, left ventricular end diastolic volume; LVESV, left ventricular end systolic volume; LA, left atrial; BSA, body surface area; GLS, global longitudinal strain; E, passive ejection of left atrium; A, active atrial contraction; e', diastolic annular velocity.

### The IndEcho cohorts

The IndEcho study is a longitudinal observational study that recalled participants from two birth cohorts in New Delhi (NDBC) and Vellore (VBC), India. These are prospective population-based cohorts originally set up in 1969–1972 to collect data on birth outcomes and infant mortality. The original cohorts included 18,700 singleton live births recruited from urban (the city of New Delhi and Vellore town) and rural (Vellore only) populations. Both studies collected longitudinal data on the growth of the children. In an adult follow-up at the age of 26–33 years, data was collected on a range of classical CVD risk factors. Details of both cohorts and previous rounds of follow-up are presented elsewhere (9–11). In the current study, men and women aged 43–46 years underwent echocardiography and cardiometabolic phenotyping. The sampling frame included all the men and women who took part in the 26–33 year follow-up in both cohorts (*N* = 1,526 in New Delhi and *N* = 2,218 in Vellore). The study was approved by the Health Ministry Steering, Government of India, institutional ethics committees of the participating centres and the University of Southampton, UK.

### Clinical assessment

All participants, after consenting to participate, underwent clinical assessment using standardised protocols. Assessment included anthropometry (height, weight, waist and hip circumferences), biochemistry (fasting glucose and an oral glucose tolerance test, fasting plasma insulin and serum lipids, urinary albumin-creatinine ratio as a measure of microalbuminuria), assessments of life-style behaviour (tobacco and alcohol consumption, physical activity) and socio-economic status [using the standard of living index (SLI)], detailed echocardiographic assessment of LV dimensions and function and a 12-lead ECG with Minnesota coding. SLI was calculated as a composite score based on education, type of housing, household amenities, and material possessions (12).

### Echocardiographic measurements

#### Cardiac size

LV internal dimensions in systole (LVIDs) and diastole (LVIDd), inter-ventricular septal thickness (IVS) and posterior wall thickness (PWT) were measured by M-mode using the leading-edge method. Relative wall thickness (RWT) was computed using the formula [(2 X PWT)/LVIDd]. Fractional shortening (FS) was calculated as the ratio of the difference between LVIDd and LVIDs to LVIDd. LV end-diastolic volume (LVEDV) and LV end-systolic volume (LVESV) were measured from trans-thoracic 2D recordings using the modified Simpson's biplane method, indexed to body surface area (BSA). LV mass was derived using the area-length method by contouring the epicardial and endocardial cross-sectional areas at the papillary muscle level along with LV length measured from the from the mitral annulus to the apex.

#### Systolic function

From the 2D-recordings of LVEDV and LVESV, LV ejection fraction (LVEF) was calculated as [(LVEDV-LVESV)/LVEDV]. Global longitudinal strain (GLS) was computed by speckle tracking from the acquired 2-D; 2-, 3- and 4-chamber and long axis views using the in-built QLAB software in the CX50 echo machine. Measurements were acquired offline in standard grey-scale with a frame rate of at least 40 frames per minute.

#### Diastolic function

Myocardial velocities were measured using tissue-doppler imaging at septal and lateral mitral annulus. Trans-mitral inflow parameters were measured in early and late diastole in the apical 4C-view. Peak mitral flow velocities (early E and late A) of LV filling, the deceleration time (DT) of the E-wave, and the ratio of E/A was calculated. Peak mitral annular velocity (e') was measured in the apical 4C-view at the septal and lateral mitral annulus, and the average e' was calculated from two (septal and lateral) mitral annular segments. The E/e' ratio was derived subsequently. The LA volumes (indexed to BSA) were recorded in both 2C- and 4C-views and the average of these values was calculated. Right ventricular systolic pressure (RVSP) was determined from peak TR jet velocity, using the simplified Bernoulli equation, and combining this value with an estimate of the RA pressure: RVSP = 4(*V*)^2 ^+ RA pressure, where *V* is the peak velocity (in meters per second) of the tricuspid valve regurgitant jet. The RA pressure was estimated using the IVC diameter and collapsibility using criteria described by the ASE.

#### Outcome definitions

Type 2 diabetes (T2DM) was diagnosed from the fasting and 120 min glucose concentration after a 75 g oral glucose load using WHO criteria (13). Participants were also defined as having T2DM if they had a prior diagnosis of the condition managed by diet and/or medication. Hypertension was defined as a systolic blood pressure ≥140 mmHg or diastolic pressure ≥90 mmHg or being on prescribed treatment for hypertension. A past history of myocardial infarction was defined as a history of a heart attack diagnosed by their treating physician, and/or the presence of major *Q* waves on the ECG. Overweight was defined as BMI > 25–<30 kg/m^2^ and obesity as BMI > 30 kg/m^2^.

Cardiac chamber dimensions were calculated according to ASE/EACVI guidelines. LV remodelling and hypertrophy were categorised based on LV mass (BSA indexed) and RWT as concentric remodelling (LV mass ≤102 g in men/≤88 g in women and RWT >0.42), concentric hypertrophy (LV mass >102 g in men/>88 g in women and RWT > 0.42), and eccentric hypertrophy (LV mass >102 g in men/>88 g in women and RWT ≤ 0.42) (8).

Systolic dysfunction was based on EF and GLS values. Low EF was defined as <52% in men and <54% in women (12). GLS was categorised as normal, borderline or abnormal if strain measurements were <−17%, between −17 and −15%, or >−15% respectively (8).

Diastolic dysfunction was defined only if the EF was normal according to the criteria proposed by the joint ASE/European Association of Echocardiography guidelines (14). Diastolic dysfunction was defined if more than two of the following four abnormalities were present: (1) septal (medial) e' <7 cm/sec or lateral e' <10 cm/sec; (2) E/e' >14; (3) LA volume >34 ml/m^2^; and (4) TRmax >2.8 m/sec. If exactly two of these abnormalities were present, diastolic function was defined as indeterminate.

#### Quality control and reproducibility of measurements

The quality of images was assessed by cardiologists. Images deemed “good” or “fair” were included in the analysis. “Good” was defined as adequate images with clear endocardial and epicardial boundaries, allowing clear measurements of the IVS, PW, LVID in PLAX and for calculating LV volumes and LVEF from short axis and apical projections. “Fair” was defined as adequate apical views allowing the tracing of the endocardial boundary for apical 2- and 3-chamber views and adequate calculations of LVEF, but inadequate short axis views, precluding optimal wall motion and LV area estimates, or clear measurements for IVS, PW and LVID in only one view. Unsatisfactory images were defined as those in which it was not possible to trace endocardial boundaries and therefore to calculate reliable LVEF, LV areas or wall motion abnormalities, and/or M-mode projections inadequate to measure IVS, PW or LVID.

To quantify the reproducibility of measurements, the intra-observer variation of selected echocardiographic measurements (pre-specified indices of LV systolic function, LV diastolic function and LV mass) was analysed in two independent readings from the recordings of a subset of 20 randomly selected participants.

### Statistical analysis

**Figure 1:**
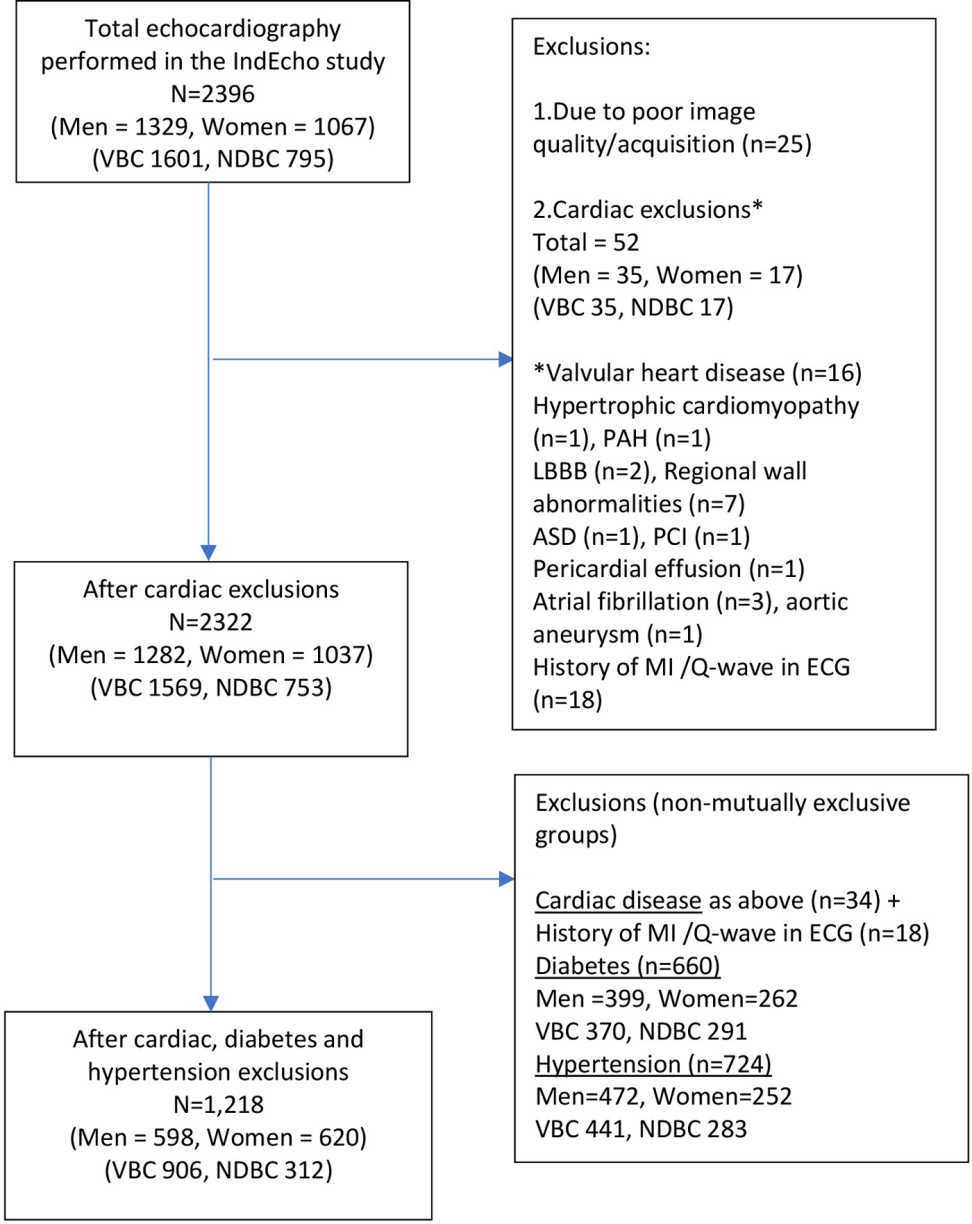
Flow diagram of participants who underwent echocardiographic measurements in the IndEcho study.

Intra-observer reproducibility was assessed by intraclass correlation coefficients (ICC) with 95% CI, interpreted as follows: ICC < 0.5 as poor reproducibility; ICC, 0.5–<0.75 as moderate; ICC, 0.75–0.9 as good; and >0.9 as excellent (15). Agreement was measured by Bland-Altman (BA) analysis and reported as the mean difference with 95% limits of agreement. Descriptive data are presented stratified by cohort and sex, because of differences in the frequencies of underlying co-morbidities such as diabetes, hypertension and heart disease. Continuous variables are presented as mean and standard deviation (SD) or median and interquartile range (IQR) depending on the skewness of the data. Frequency (percentages, %) were calculated for categorised variables. We present data for the whole sample, excluding individuals with known major cardiac disease likely to distort the echocardiographic measurements (valve disease, septal defects, hypertrophic cardiomyopathy, and pericardial effusion) (Table 2) and separately for a “healthy” sub-set free of hypertension, diabetes or a history of myocardial infarction (Table 3). A study flowchart and exclusions are presented in Figure 1. All analyses were performed using the STATA statistical package, version IC/16.0 (StataCorp LLC, United States). A *p*-value of <0.05 was considered statistically significant.

**Table 2:** Descriptive data stratified by cohort (NDBC and VBC) free of major structural cardiac disease.

Variable units	DELHI				VELLORE				*p*-value between sexes
	MALES		FEMALES		MALES		FEMALES		
	*N*	Mean SD/Median IQR^†^/*N* %	*N*	Mean SD/Median IQR^†^/*N* %	*N*	Mean SD/Median IQR^†^/*N* %	*N*	Mean SD/Median IQR^†^/*N* %	
Clinical characteristics									
Age (years)	460	46.1 (1.13)	293	46.1 (1.06)	824	45.9 (1.11)	745	46.0 (1.14)	0.034
Weight (kg)	457	80.0 (15.1)	289	70.7 (13.0)	824	68.5 (13.4)	745	62.5 (13.1)	<0.001
Height (cm)	456	169.8 (6.6)	290	154.9 (5.7)	824	167.2 (6.4)	745	154.6 (5.7)	<0.001
BMI (kg/m^2^)	456	27.7 (4.7)	289	29.4 (5.1)	824	24.4 (4.2)	745	26.1 (5.1)	<0.001
Body surface area (m^2^)	456	1.91 (0.18)	289	1.69 (0.15)	824	1.76 (0.18)	745	1.60 (0.16)	<0.001
Systolic blood pressure (mmHg)	456	129.4 (16.6)	289	121.3 (17.6)	824	130.5 (19.2)	745	125.0 (18.5)	<0.001
Diastolic blood pressure (mmHg)	456	87.9 (10.9)	289	82.5 (11.2)	824	79.6 (13.9)	745	75.7 (11.3)	<0.001
Pulse (bpm)	456	82.6 (10.6)	289	85.5 (10.0)	824	80.8 (12.0)	745	85.4 (11.2)	<0.001
SLI score	460	41.6 (5.1)	293	41.0 (5.6)	824	29.5 (6.6)	745	28.8 (6.7)	
Tobacco use									
Never		297 (65.4)		287 (99.0)		499 (60.6)		698 (93.7)	
Ex-user		34 (7.5)		0 (0.0)		69 (8.4)		5 (0.7)	<0.001
Current user		123 (27.1)		3 (1.0)		256 (31.1)		42 (5.6)	
Alcohol consumption									
None		179 (38.9)		281 (95.9)		321 (39.0)		745 (100.0)	
Mild		207 (45.0)		12 (4.1)		327 (39.7)		0 (0.0)	<0.001
Moderate		49 (10.7)		0 (0.0)		104 (12.6)		0 (0.0)	
Heavy		25 (5.4)		0 (0.0)		72 (8.7)		0 (0.0)	
CARDIAC SIZE									
LVIDd (mm)	434	48.8 (6.1)	272	45.4 (5.7)	824	45.6 (4.6)	745	43.0 (4.5)	<0.001
LVIDs (mm)	434	32.3 (4.2)	272	29.8 (4.1)	824	29.3 (3.2)	745	27.5 (2.9)	<0.001
Fractional shortening	434	0.3 (0.05)	272	0.3 (0.06)	824	0.4 (0.04)	745	0.4 (0.04)	0.019
PWT (mm)^†^	434	9.0 (8.3, 10.0)	272	8.7 (7.7, 9.0)	824	9.0 (8.3, 9.3)	745	8.7 (8.0, 9.0)	<0.001
Septal thickness (mm)^†^	434	10.0 (9.0, 10.7)	272	9.0 (8.3, 10.0)	824	10.0 (9.0, 10.3)	745	9.3 (9.0, 10.0)	<0.001
Relative wall thickness	434	0.4 (0.06)	272	0.4 (0.10)	824	0.4 (0.06)	745	0.4 (0.06)	0.083
LVEDV 4c (ml)	383	59.3 (16.1)	231	51.3 (13.3)	824	68.2 (57.7, 82.6)	745	58.0 (49.4, 70.0)	<0.001
LVESV 4c (ml)	383	21.4 (6.5)	231	18.4 (5.1)	824	23.5 (19.7, 28.4)	745	20.1 (17.0, 23.7)	<0.001
LV mass absolute (g)^†^	348	128.5 (109.0, 152.0)	212	109.0 (94.3, 130.0)	824	117.0 (99.1, 135.0)	745	99.3 (85.0, 114.0)	<0.001
LV mass^i^ indexed for BSA (g/m^2^)^†^	345	68.0 (58.8,79.4)	208	63.9 (56.4, 75.3)	824	65.8 (57.7, 75.9)	745	61.6 (54.6, 70.3)	<0.001
Categories of LV re-modelling/hypertrophy									
None (*n*, %)		244 (73.9)		139 (69.5)		573 (69.5)		471 (63.2)	0.007
Concentric remodelling (*n*, %)		70 (21.2)		38 (19.0)		231 (28.0)		247 (33.2)	
Concentric hypertrophy (*n*, %)		3 (0.9)		6 (3.0)		8 (1.0)		10 (1.3)	
Eccentric hypertrophy (*n*, %)		13 (3.9)		17 (8.5)		12 (1.5)		17 (2.3)	
SYSTOLIC FUNCTION									
LV ejection fraction (%) ^†^	383	64.5 (61.6, 67.3)	231	64.9 (61.7, 67.2)	824	65.4 (63.2, 67.3)	745	65.7 (63.6, 67.5)	0.031
Low LV ejection fraction (*n*, %)		7 (1.8)		4 (1.7)		7 (0.9)		2 (0.3)	0.184
Global Longitudinal Strain (%)	452	−18.0 (3.4)	289	−18.3 (2.7)	824	−19.1 (2.2)	744	−19.7 (2.2)	<0.001
Categories of GLS									
Normal (*n*, %)		358 (79.2)		238 (82.4)		707 (85.8)		672 (90.3)	0.003
Borderline (*n*, %)		75 (16.6)		48 (16.6)		83 (10.1)		52 (7.0)	
Abnormal (*n*, %)		19 (4.2)		3 (1.0)		34 (4.1)		20 (2.7)	
DIASTOLIC FUNCTION additionally excluded people in atrial fibrillation									
Mitral - E (cm/sec)^†^	459	71.8 (63.4, 81.2)	293	75.8 (68.6, 85.0)	824	74.4 (64.8, 84.0)	741	78.8 (69.8, 89.5)	<0.001
Mitral - A (cm/sec)^†^	459	64.0 (57.2, 72.8)	293	68.2 (61.8, 75.4)	824	60.4 (53.1, 67.6)	741	64.6 (57.0, 73.7)	<0.001
Mitral E/A^†^	459	1.1 (1.0, 1.3)	293	1.1 (1.0, 1.3)	824	1.2 (1.1, 1.4)	741	1.2 (1.1, 1.4)	0.983
Deceleration time (ms)	459	147.6 (28.4)	293	147.1 (31.6)	824	147.8 (31.1)	741	143.1 (31.0)	0.003
Mitral E/average e’^†^	457	7.4 (6.5, 8.4)	293	7.8 (6.8, 9.2)	824	7.9 (6.9, 9.2)	741	8.6 (7.4, 9.7)	<0.001
LA volume^i^ (ml/m^2^) - 4c^†^	380	10.0 (7.7, 11.8)	227	10.9 (8.2, 12.6)	824	11.6 (9.7, 13.6)	744	11.9 (10.1, 14.1)	<0.001
Average LA volume^i^ (ml/m^2^)^†^	352	9.8 (8.4, 11.6)	206	10.5 (8.8, 12.6)	824	11.4 (9.7, 13.1)	744	11.9 (10.4, 13.8)	<0.001
TR max m/sec	298	154.6 (35.8)	179	156.4 (36.7)	822	174.0 (39.0)	744	179.6 (37.9)	<0.001
Categories of LV diastolic dysfunction participants with normal LV ejection fraction only									
None (*n*, %)		295 (78.5)		182 (80.2)		809 (99.0)		737 (99.2)	0.038
Indeterminate (*n*, %)		81 (21.5)		45 (19.8)		8 (1.0)		6 (0.8)	
Dysfunction (*n*, %)		0		0		0		0	
Co-morbidities									
Diabetes (*n*, %)		187 (40.7)		104 (35.5)		212 (25.7)		158 (21.2)	0.006
Hypertension (*n*, %)		204 (44.4)		79 (27.0)		268 (32.5)		173 (23.2)	<0.001
Myocardial infarction^ (*n*, %)		4 (0.9)		0 (0.0)		5 (0.6)		0 (0.0)	NA
Atrial fibrillation (*n*, %)		–		–		0 (0.0)	** **	1 (0.1)	NA
Overweight (BMI ≥ 25 kg/m^2^), *n* (%)		200 (43.9)		112 (38.8)		291 (35.3)		256 (34.4)	<0.001
Obesity (BMI ≥ 30 kg/m^2^), *n* (%)		126 (27.6)		124 (42.9)		77 (9.3)		168 (22.5)	<0.001

LV, left ventricle; LVIDD, LV internal diameter in diastole; LVISD, LV internal diameter in systole; PWT, posterior wall thickness; GLS, global longitudinal strain; E, passive ejection of left atrium; A, active atrial contraction; e’, diastolic annular velocity.

We excluded 52 participants (NDBC *N* = 17, VBC *N* = 35) due to major cardiac abnormalities likely to distort the echocardiographic measurements, including valvular heart disease, septal defects, hypertrophic cardiomyopathy and pleural effusion.

^ MI defined as history of MI in both cohorts.

^†^ denotes median (interquartile range).

**Table 3:** Descriptive data stratified by cohort, further excluding participants with diabetes, hypertension or a history of MI.

Variable units	DELHI				VELLORE				*p*-value between sexes
	MALES		FEMALES		MALES		FEMALES		
	*N*	Mean SD/Median IQR^†^/*N* %	*N*	Mean SD/Median IQR^†^/*N* %	*N*	Mean SD/Median IQR^†^/*N* %	*N*	Mean SD/Median IQR^†^/*N* %	
Clinical characteristics									
Age (years)	164	46.2 (1.2)	148	46.0 (1.0)	434	45.8 (1.1)	472	46.0 (1.2)	0.349
Height (cm)	163	169.8 (5.9)	148	154.1 (5.9)	434	65.0 (12.8)	472	59.9 (12.2)	<0.001
Weight (kg)	163	77.3 (14.6)	148	69.7 (13.1)	434	166.9 (6.4)	472	154.7 (5.9)	<0.001
BMI (kg/m^2^)	163	26.8 (4.6)	148	29.4 (5.4)	434	23.3 (4.0)	472	25.0 (4.8)	<0.001
Body surface area (m^2^)	163	1.88 (0.18)	148	1.68 (0.15)	434	1.72 (0.17)	472	1.57 (0.16)	<0.001
Systolic blood pressure (mmHg)	163	118.9 (9.6)	146	113.5 (11.0)	434	120.2 (10.5)	472	116.9 (12.1)	<0.001
Diastolic blood pressure (mmHg)	163	80.0 (6.2)	146	77.4 (7.7)	434	72.4 (9.5)	472	71.2 (8.5)	<0.001
Pulse (bpm)	163	80.0 (9.6)	146	84.8 (9.7)	434	77.9 (11.0)	472	83.6 (10.0)	<0.001
SLI score	164	41.7 (4.1)	147	41.1 (4.6)	434	28.7 (6.6)	472	28.1 (6.9)	<0.001
Tobacco use									
Never		114 (69.5)		144 (98.0)		261 (60.1)		436 (92.4)	
Ex-user		7 (4.3)		0 (0.0)		25 (5.8)		4 (0.9)	<0.001
Current user		43 (26.2)		3 (2.0)		148 (34.1)		32 (6.8)	
Alcohol consumption									
None		72 (43.9)		141 (95.9)		192 (44.2)		472 (100.0)	
Mild		75 (45.7)		6 (4.1)		169 (38.9)		0 (0.0)	<0.001
Moderate		14 (8.5)		0 (0.0)		51 (11.8)		0 (0.0)	
Heavy		3 (1.8)		0 (0.0)		22 (5.1)		0 (0.0)	
CARDIAC SIZE									
LVIDd (mm)	157	48.4 (5.8)	139	45.1 (5.1)	434	45.2 (4.4)	472	42.8 (4.3)	<0.001
LVIDs (mm)	157	32.2 (4.1)	139	29.6 (3.5)	434	29.0 (2.9)	472	27.4 (2.9)	<0.001
Fractional shortening	157	0.3 (0.04)	139	0.3 (0.05)	434	0.4 (0.04)	472	0.4 (0.04)	0.052
PWT (mm)^†^	157	9.0 (8.0, 9.3)	139	8.0 (7.0, 9.0)	434	9.0 (8.3, 9.0)	472	8.7 (8.0, 9.0)	<0.001
Septal thickness (mm)^†^	157	9.5 (9.0, 10.2)	139	9.0 (8.0, 10.0)	434	10.0 (9.0, 10.3)	472	9.0 (9.0, 10.0)	<0.001
Relative wall thickness	157	0.4 (0.06)	139	0.4 (0.12)	434	0.4 (0.05)	472	0.4 (0.05)	0.075
LVEDV 4c (ml)^†^	142	55.8 (45.8, 64.3)	115	49.1 (41.8, 58.0)	434	67.3 (57.0, 81.1)	472	58.0 (50.6, 69.7)	<0.001
LVESV 4c (ml)^†^	142	19.1 (15.7, 21.8)	115	17.4 (14.1, 21.5)	434	23.1 (19.1, 27.9)	472	20.1 (17.0, 23.8)	<0.001
LV mass absolute (g)^†^	127	123.0 (105.0, 145.0)	108	104.0 (92.8, 124.5)	434	112.0 (95.5, 127.0)	472	95.3 (82.1, 111.0)	<0.001
LV mass^i^ indexed for BSA (g/m^2^)^†^	126	65.4 (57.3, 75.2)	108	62.6 (56.5, 75.5)	434	65.0 (57.2, 74.2)	472	60.9 (54.2, 70.1)	<0.001
Categories of LV re-modelling/hypertrophy									
None (*n*, %)		97 (80.2)		73 (70.9)		319 (73.5)		318 (67.4)	0.023
Concentric remodelling (*n*, %)		18 (14.9)		17 (16.5)		110 (25.4)		142 (30.1)	
Concentric hypertrophy (*n*, %)		2 (1.7)		3 (2.9)		0 (0.0)		4 (0.9)	
Eccentric hypertrophy (*n*, %)		4 (3.3)		10 (9.7)		5 (1.2)		8 (1.7)	
SYSTOLIC FUNCTION									
LV ejection fraction (%)^†^	142	65.2 (62.6, 67.8)	115	64.9 (61.0, 67.6)	434	65.7 (63.6, 67.5)	472	65.9 (63.8, 67.7)	0.633
Low LV ejection fraction (*n*, %)		2 (1.4)		3 (2.6)	434	0 (0.0)	472	2 (0.4)	0.425
Average GLS	162	−17.9 (4.3)	146	−18.3 (3.4)	434	−19.6 (2.2)	472	−20.2 (2.1)	<0.001
Categories of GLS									
Normal (*n*, %)		130 (80.3)		125 (85.6)		393 (90.6)		443 (93.9)	0.045
Borderline (*n*, %)		27 (16.7)		20 (13.7)		29 (6.7)		21 (4.5)	
Abnormal (*n*, %)		5 (3.1)		1 (0.7)		12 (2.8)		8 (1.7)	
DIASTOLIC FUNCTION additionally excluded people in atrial fibrillation									
Mitral - E (cm/sec)^†^	164	69.6 (62.6, 78.1)	148	75.3 (67.0, 82.2)	434	74.3 (65.0, 83.6)	470	78.3 (69.4, 87.8)	<0.001
Mitral - A (cm/sec)^†^	164	60.7 (55.2, 67.6)	148	66.5 (59.4, 73.7)	434	56.7 (50.8, 64.2)	470	62.4 (55.3, 70.0)	<0.001
Mitral E/A^†^	164	1.2 (1.1, 1.3)	148	1.2 (1.0, 1.3)	434	1.3 (1.2, 1.5)	470	1.3 (1.2, 1.4)	0.084
Deceleration time (ms)	164	149.4 (27.8)	148	150.9 (30.2)	434	150.1 (31.7)	470	145.4 (28.9)	0.045
Mitral E/average e’^†^	162	7.1 (6.3, 8.2)	148	7.6 (6.7, 8.6)	434	7.4 (6.5, 8.6)	470	8.3 (7.1, 9.4)	0.002
LA volume^i^ (ml/m^2^) - 4c^†^	134	9.8 (8.1, 11.6)	106	10.3 (8.6, 12.8)	434	11.7 (9.6, 13.7)	472	12.0 (10.2, 14.3)	0.006
Average LA volume^i^ (ml/m^2^)^†^	133	9.5 (8.1, 11.3)	105	10.4 (8.7, 12.6)	434	11.4 (9.8, 13.2)	472	12.1 (10.5, 14.0)	<0.001
TR max m/sec	111	157.5 (34.0)	84	159.9 (39.0)	434	178.6 (37.5)	472	182.6 (36.4)	0.028
Categories of LV diastolic dysfunction participants with normal LV ejection fraction only									
None (*n*, %)		116 (82.9)		86 (76.8)		431 (99.3)		468 (99.6)	0.932
Indeterminate (*n*, %)		24 (17.1)		26 (23.2)		3 (0.7)		2 (0.4)	
Dysfunction (*n*, %)		0 (0.0)		0 (0.0)		0 (0.0)		0 (0.0)	
Co-morbidities									
Overweight (BMI ≥ 25 kg/m^2^), *n* (%)		72 (42.9)		57 (38.8)		144 (30.9)		144 (30.5)	<0.001
Obesity (BMI ≥ 30 kg/m^2^), *n* (%)		35 (21.5)		61 (41.5)		20 (4.6)		79 (16.7)	<0.001

LV, left ventricle; LVIDD, LV internal diameter in diastole; LVISD, LV internal diameter in systole; PWT, posterior wall thickness; GLS, global longitudinal strain; E, passive ejection of left atrium; A, active atrial contraction; e’, diastolic annular velocity.

^†^denotes median (interquartile range).

## Results

Echocardiographic assessment was performed on 1,329 men and 1,067 women. Of these, data from 25 participants in New Delhi were excluded, either because of a poor echo window (*n* = 11) or the scans were of unsatisfactory quality (*n* = 14). Fifty-two participants were excluded because of major pre-existing cardiac disease. The main analysis included a total of 2,322 individuals (753 in NDBC and 1,569 in VBC) (Figure 1). Their clinical characteristics and echocardiographic measurements are summarised in Table 2. Participants from both cohorts were of similar age. The BMI and BSA were higher in the NDBC compared with the VBC, as were the prevalence of other co-morbid conditions such as overweight, obesity, diabetes and hypertension.

The cardiac size parameters (LV mass, LV internal diameters, PWT and LV volumes) were generally larger in men compared with women in both cohorts, and higher in the NDBC compared with the VBC. Indexation for BSA did not alter the gender differences in LV mass. Based on current threshold definitions (8), about 30% of individuals met the diagnostic criteria for concentric remodelling and <3% had hypertrophy.

The average GLS was more negative among VBC participants (suggesting better LV systolic function) compared with NDBC participants. The mean LV ejection fraction of the IndEcho participants was well within the normal range for Indians reflecting very small variability in chamber dimensions. The proportion with low LV ejection fraction was <3% in the cohort. The trans-mitral flow velocities (E, A and E/A) and tissue doppler velocity (E/e') were comparable between both cohorts, and women generally had higher flow velocity values, but similar E/A ratio and mitral E-deceleration time compared with men.

To create the healthy sub-sample, we examined the distribution of echocardiographic outcomes in 1,215 participants (men 598, women 617) by excluding participants with diabetes, hypertension or a history of myocardial infarction (Table 3). This healthy group had smaller cardiac dimensions, more negative GLS values and similar LV filling pressures suggesting that underlying comorbid illnesses exert a pronounced effect on cardiac geometry and systolic function.

To further analyse the effect of lifestyle, we present data for the VBC participants stratified by area of residence (rural/urban) (Supplementary Table S1). The urban residents had higher SLI scores and higher tobacco and alcohol consumption. They also had higher BMI, higher BSA and a higher prevalence of diabetes, hypertension and MI. Cardiac size was similar between between rural and urban groups**.** A higher proportion of individuals from urban residence had LV remodelling and hypertrophy. A significant difference in GLS (*p* < 0.001) was observed between both groups and rural residents had higher proportion of abnormal GLS, particularly women (7.6%).

### Intra-observer variability

**Figure 2:**
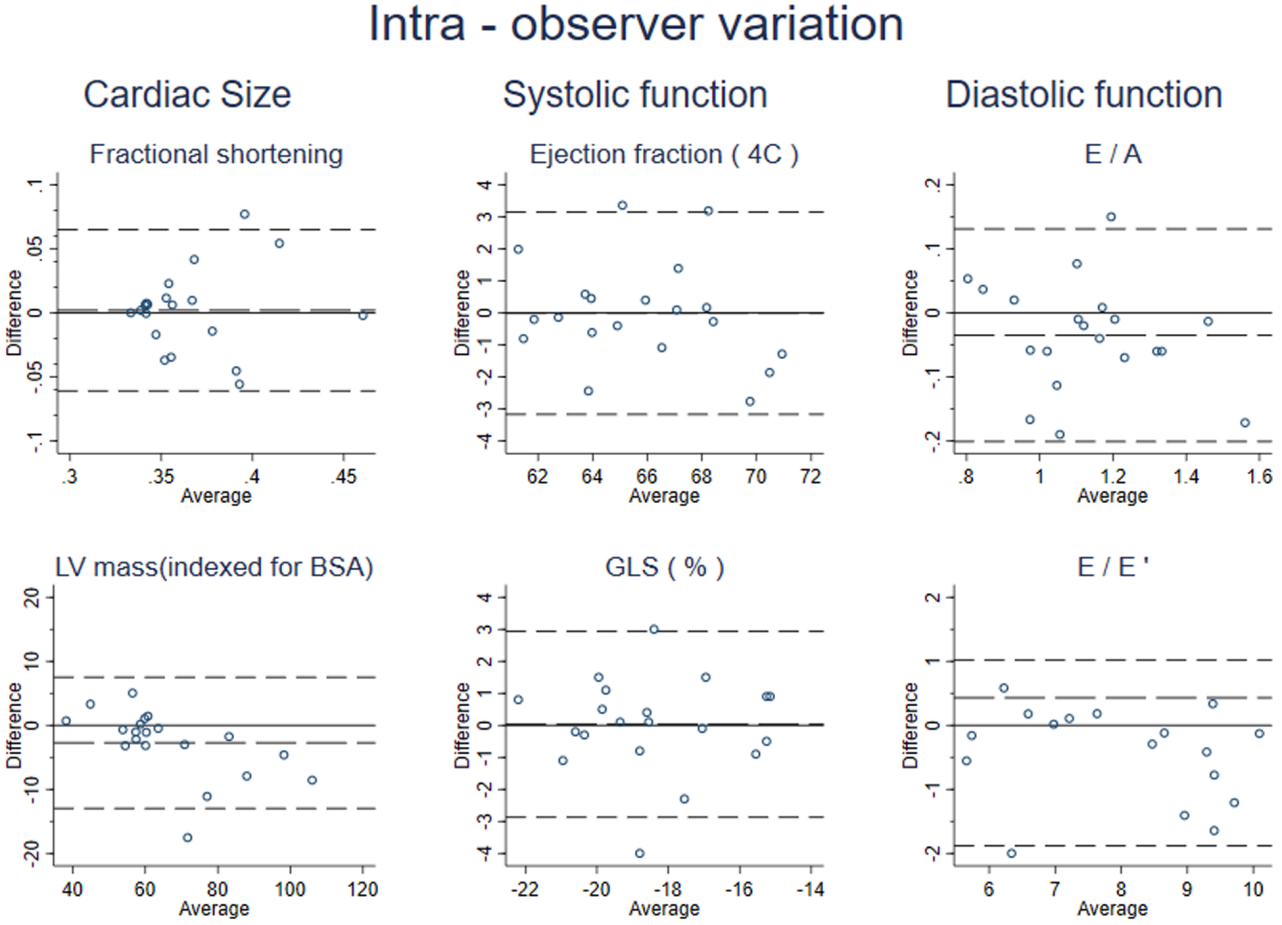
Bland–Altman plots for selected echocardiographic variables.

The Intra-observer repeatability of conventional echocardiographic measures was good or excellent in our study (Table 4). The mean difference [95% limit of agreement (LOA)] and ICC (95% CI) were −2.735 (−12.980, 7.510) and 0.971 (0.915, 0.989) for LV mass indexed for BSA. The corresponding values for other cardiac size, and systolic and diastolic function parameters are shown in Table 4. The LoA and the BA plots shown in Figure 2 demonstrate minimal bias in the data across the range of values.

**Table 4:** Intra-observer variability of echocardiographic measurements.

Variables	*n*	Correlation	Bias (SD)	LoA	CV	ICC (95% CI)
Cardiac size						
LVIDD (mm)	20	0.939	−0.033 (1.841)	(−3.641, 3.574)	2.3	0.967 (0.916, 0.987)
LVISD (mm)	20	0.906	−0.108 (1.524)	(−3.095, 2.879)	3.1	0.950 (0.874, 0.980)
PWd (mm)	20	0.701	0.442 (0.661)	(−0.853, 1.737)	4.5	0.767 (0.320, 0.913)
LVEDV (4c) (ml)	20	0.987	−2.382 (2.618)	(−7.514, 2.750)	2.7	0.988 (0.909, 0.996)
LVESV (4c) (ml)	20	0.969	−0.778 (1.468)	(−3.655, 2.098)	4.1	0.980 (0.939, 0.993)
LV mass (2D) (g)	20	0.982	−4.675 (8.381)	(−21.102, 11.752)	3.9	0.983 (0.947, 0.994)
LV mass^i^ (indexed for BSA, 2D) (g/m^2^)	20	0.969	−2.735 (5.227)	(−12.980, 7.510)	3.9	0.971 (0.915, 0.989)
Systolic function						
LV ejection fraction	20	0.868	−0.014 (1.611)	(−3.171, 3.143)	1.3	0.929 (0.821, 0.972)
Average GLS (%)	20	0.77	0.030 (1.482)	(−2.874, 2.934)	4.1	0.876 (0.683, 0.951)
Diastolic function						
E (cm/sec)	20	0.98	−2.372 (2.822)	(−7.904, 3.160)	3.1	0.983 (0.899, 0.995)
A (cm/sec)	20	0.893	−0.250 (4.264)	(−8.608, 8.108)	3.2	0.944 (0.857, 0.978)
E/A	20	0.911	−0.035 (0.085)	(−0.201, 0.131)	4.4	0.946 (0.858, 0.979)
Deceleration time (ms)	20	0.958	1.442 (9.260)	(−16.707, 19.590)	3.5	0.976 (0.940, 0.990)
Lateral e’ (cm/sec)	17	0.953	0.309 (0.654)	(−0.974, 1.591)	3.9	0.966 (0.899, 0.988)
Medial e’ (cm/sec)	16	0.916	0.152 (0.571)	(−0.966, 1.271)	3.8	0.953 (0.869, 0.983)
LA volume (ml/m^2^) - 2c	20	0.964	0.737 (1.051)	(−1.322, 2.796)	3.8	0.974 (0.891, 0.991)
LA volume (ml/m^2^) - 4C	20	0.978	0.138 (1.047)	(−1.913, 2.190)	3.5	0.988 (0.969, 0.995)

LoA, limits of agreement; CV, co-efficient of variation; ICC (95% CI), inter-class correlation (95% confidence interval).

## Discussion

The current study presents comprehensive echocardiographic data in over 2,300 individuals aged 43–50 years and for a “healthy” sub-set apparently free from major cardiac disease, hypertension or diabetes, as well as presenting the reproducibility of echocardiographic measurements.

Our results confirm expected sex differences, heterogeneity across sites and rural/urban differences in cardiac dimensions and function. Overall, the cardiac dimensions and functional measurements align with other previously published literature among Indians (16–19). The NDBC participants generally had larger ventricular internal dimensions than VBC participants, including indexed LV mass, which could be due to the higher prevalence of underlying hypertension and higher BMI in this cohort. Systolic function was characterised by similar EF between both groups, while the NDBC participants demonstrated lower (less negative) GLS, possibly denoting a sizeable proportion with undetected subclinical heart disease that warrants further investigation. The heterogeneity between sites could possibly be driven by lifestyle differences and other risk factors. Overall, men had higher LV volume and lower systolic function compared with women. Consistent site-specific and sex differences were observed even after exclusion of individuals with diabetes and hypertension. Differences were also observed between urban and rural residents, with the former group having a higher LV mass, lower LV volumes and poor systolic function.

The Indian Normative Data of Echocardiography Analyzed (INDEA) (17) and World Alliance Societies of Echocardiography (WASE) (19) studies are the largest published studies that provide reference ranges for echo parameters in Asian Indians. The reference values of the WASE study that included 2008 individuals across 15 countries including India report variation in normative values across countries and between sexes. Our values fall within the most commonly used reference range provided by INDEA (17), WASE (Indian reference) (19) and ASE/EACVI (Western range) (8) (Supplementary Table S2). Similarly, cardiac dimensions (LV mass, LVEDV and RWT) in our study are comparable to other native Indian reports (16, 20–22), but smaller than the western population. A retrospective singe-centred study from central India by Sullere et al., reported that Indians have similar or even larger cardiac dimensions and volumes than those of Western origin (20). Ethnic based differences in LV size are well recognised (23, 24) and could be partly attributed to the differences in body composition between the ethnic groups.

Our data showed a higher prevalence of LV re-modelling and lower annular velocities than in US/European studies. The LV remodelling could reflect the high prevalence of underlying hypertension in this cohort and may contribute to increased CVD risk in this ethnic group, rather than hypertrophy. However, systolic (EF and GLS values) and diastolic function are comparable to other ethnic groups despite the smaller cardiac volume (18, 19, 25).

Comparative data on the parameters used to define diastolic function are less readily available; the INDEA study reported E, A, and E/e' values for a very small sub-set of the participants (*N* = 100) whose age was not defined (medial e' 10.2 cm/s in men, 10.1 cm/s in women; average E/e' 7.4 and 7.7; LA volume indexed 17.8 and 18.6 ml/m^2^; TR max 134 and 135 cm/s). The diastolic function in our study participants was well within the global (ASE/EACVI) and INDEA thresholds (8, 17).

The sex differences reported in our study have also been recognised previously (16, 17, 26). Consistent demonstration of sex differences in various ethnic groups may suggest that women generally have better LV adaptation to CVD compared with men (27, 28). The heterogeneity observed between cohorts within India as well as rural-urban differences could be attributed to burden of underlying co-morbid illnesses such as diabetes, obesity and hypertension in the community as well as differences in life-style factors within a country.

### Strengths and limitations

Strengths were that the IndEcho study is one of the largest population-based echocardiographic studies from India and adds to the existing Echo-normative data in Indians. This resource is additionally valuable because of the past serially collected data on childhood growth and cardiometabolic risk markers from young adulthood, which will enable us to analyse these as longitudinal predictors of LV structure and function in future. The echocardiographic measurements demonstrated good to excellent repeatability. Due to the cohort design, our study sample covered a narrow age range, and therefore our findings in relation to cardiac structure and function may not be generalisable to other ages. The INDEA study, which included three age-groups (<35, 36–55 and ≥56 years) found little change in cardiac dimensions or systolic function with age (14). However, there are well-documented changes in LV diastolic function with age (9). Few participants (*n* = 25) had to be excluded completely due either to failure to obtain a suitable echo window, or poor image quality, most commonly associated with obesity, leading to some loss of study population. However, the proportion lost in IndEcho was similar to that in other studies (14% in the INDEA study). Since there are no widely accepted reference standards for LV remodelling/hypertrophy, systolic and diastolic function, among Asian Indians, our definitions were based on the US and European cut-offs which may not be ideal given the differences in cardiac size and could underestimate the prevalence of LV remodelling/hypertrophy in this cohort, a potential pitfall in applying western normal range values to Indian population.

## Conclusions

In this large population-based analysis of cardiac structure and function in mid-life, our results confirm that healthy Asian Indians have smaller cardiac chamber volumes and dimension with similar systolic and diastolic function compared with published data from Europeans and white Caucasians. These reinforce the need for ethnic-specific and age-appropriate reference data to diagnose CVD as well as optimise clinical management. The high prevalence of overweight, obesity, diabetes, hypertension or a history of MI in these cohorts highlights the high prevalence of mid-life cardiometabolic disease currently in India. It is possible that inherent differences in cardiac geometry and function in Indians on a background of a high prevalence of cardiometabolic risk factors may in part contribute to premature CVD risk in this ethnic group. Future analysis will examine these risk factors, and early life growth patterns as predictors.

## Data Availability

The raw data supporting the conclusions of this article will be made available by the authors, without undue reservation.

## Member of the IndEcho study group

Bhaskar Singh, Siva Dora, Sukanya Sarma, Gupta KD (D-7 Gulmohar Park, New Delhi), Srinath Reddy (Public Health Foundation of India, New Delhi, India), Thomas V Paul (Endocrinology, Christian Medical College, Vellore), Prasanna Samuel (Biostatistics, Christian Medical College, Vellore)
