# Supplementary material for: Echocardiography protocol and cardiometabolic phenotyping in Indian birth cohorts—the IndEcho study
Source: Front Cardiovasc Med. 2023 Jul 13;10:1055454. doi: 10.3389/fcvm.2023.1055454 (PMC10372793; doi:10.3389/fcvm.2023.1055454)
Supplement: Supplementary file 1 [file Datasheet1.pdf]

Supplementary Table 1: Comparison of baseline ECHO characteristics among healthy individuals and individuals with pre-existing co-morbidities (Diabetes, Hypertension, Cardiac disease)

Variables (units)	Pre-existing comorbidities present				
	YES		NO		
	N	Mean (SD) / Median (IQR)	N	Mean (SD) / Median (IQR)	P value
Age (years)	1105	**46.06** (1.12)	1217	**45.93** (1.12)	0.005
Height (cm)	1099	**163.35** (9.11)	1216	**160.98** (9.01)	<0.001
Weight (kg)	1099	**73.31** (14.69)	1216	**65.25** (14.09)	<0.001
BMI (kg/m^2)	1098	**27.43** (4.82)	1216	**25.16** (4.95)	<0.001
Body surface area (m^2)	1098	**1.79** (0.20)	1216	**1.68** (0.19)	<0.001
SBP (mmHg)	1099	**137.70** (19.53)	1215	**117.97** (11.28)	<0.001
DBP (mmHg)	1099	**87.78** (12.45)	1215	**73.59** (9.09)	<0.001
Pulse	1099	**85.43** (11.86)	1215	**81.20** (10.64)	<0.001
**CARDIAC SIZE**					
LVIDD (mm)	1074	**46.08** (5.75)	1201	**44.69** (4.97)	<0.001
LVISD (mm)	1074	**29.84** (4.11)	1201	**28.85** (3.49)	<0.001
Fractional shortening	1074	**0.35** (0.05)	1201	**0.35** (0.04)	0.468
PWT (mm)	1074	**9** (8.33, 9.67)	1201	**8.67** (8, 9)	<0.001
Septal thickness (mm)	1074	**10** (9, 10.33)	1201	**9.33** (9, 10)	<0.001
Relative wall thickness (no units)	1074	**0.40** (0.06)	1201	**0.39** (0.07)	<0.001
LVEDV (4c) (ml)	1021	**63.60** (18.05)	1162	**62.04** (16.32)	0.132
LVESV (4c)(ml)	1021	**22.56** (6.99)	1162	**21.52** (5.96)	0.003
LV mass (2D) (g)	989	**117** (100, 140)	1140	**105** (89.9, 122.5)	<0.001
LV mass[i] (indexed for BSA, 2D) (g/m^2)	983	**66.26** (58.09, 77.35)	1139	**63.03** (55.05, 72.25)	<0.001
**Categories of LV re-modelling/hypertrophy**					
None N (%)[#]		**620** (63.98)		**807** (71.42)	
Concentric remodelling N (%)[#]		**299** (30.86)		**287** (25.40)	0.001
Concentric hypertrophy N (%)[#]		**18** (1.86)		**9** (0.80)	
Eccentric hypertrophy N (%)[#]		**32** (3.30)		**27** (2.39)	
**SYSTOLIC FUNCTION**					
LV ejection fraction (%)†	1021	**64.98** (62.59, 67.13)	1162	**65.65** (63.27, 67.64)	<0.001
Low LV ejection fraction N (%)		**14** (1.37)		**6** (0.52)	
**Global longitudinal strain (GLS)**					
Average GLS	1096	**-18.55** (2.33)	1213	**-19.43** (2.82)	<0.001
GLS 2c	1084	**-18.53** (2.63)	1202	**-19.47** (3.25)	<0.001
GLS 4c	1092	**-19.60** (2.45)	1213	**-20.28** (2.99)	<0.001
GLS long axis	969	**-17.51** (3.35)	1115	**-18.62** (3.50)	<0.001
**Categories of GLS**					
Normal N (%)[β]		**885** (80.75)		**1090** (89.86)	
Borderline N (%)[β]		**161** (14.69)		**97** (08.00)	<0.001

Abnormal N (%)$^{\beta}$		**50** (04.56)		**26** (02.14)	
**DIASTOLIC FUNCTION (additionally excluded people in atrial fibrillation)**					
E (cm/sec)	1102	**75.80** (66.40, 85.80)	1215	**75.40** (66.60, 85)	0.810
A (cm/sec)	1102	**66.63** (59.20, 75.40)	1215	**60.80** (54, 68.57)	<0.001
E/A (no units)	1102	**1.15** (0.96, 1.29)	1215	**1.25** (1.10, 01.4)	<0.001
Deceleration time (ms)	1102	**144** (121, 162)	1215	**148** (126, 169)	<0.001
Medial e' (cm/sec)	1102	**10.70** (2.36)	1213	**11.56** (2.27)	<0.001
Lateral e' (cm/sec)	1101	**7.98** (1.67)	1212	**8.70** (1.65)	<0.001
Average e' (cm/sec)	1103	**9.34** (1.82)	1213	**10.13** (1.74)	<0.001
Medial E/e' (no units)	1100	**9.71** (8.23, 11.19)	1212	**8.73** (7.57, 10.14)	<0.001
Lateral E/e' (no units)	1101	**7.08** (6.05, 8.42)	1213	**6.58** (5.64, 7.79)	<0.001
Average E/e'	1102	**8.35** (7.26, 9.75)	1213	**7.71** (6.67, 8.86)	<0.001
LA volume^{i} (ml/m^2) - 4c	1014	**11.08** (9.25, 12.9)	1161	**11.45** (9.35, 13.64)	0.029
LA volume^{i} (ml/m^2) - 2C	990	**11.09** (9.44, 13.17)	1145	**11.28** (9.38, 13.40)	0.003
Average LA volume ml/m^2	983	**11.25** (9.54, 12.75)	1143	**11.34** (9.64, 13.32)	0.035
TR max (m/sec)	943	**165.71** (39.72)	1100	**176.79** (37.73)	<0.001
**Categories of LV diastolic dysfunction (participants with normal LV ejection fraction only)***					
None N (%)$^{\gamma}$		**922** (91.56)		**1101** (95.24)	
Indeterminate N (%)$^{\gamma}$		**85** (08.44)		**55** (04.76)	0.001
Dysfunction N (%)$^{\gamma}$		**0** (0.0)		**0** (0.0)	

LV: left ventricle; LVIDD:LV internal diameter in diastole; LVISD: LV internal diameter in systole; PWT: Posterior wall thickness; GLS: Global longitudinal strain; E: passive ejection of left atrium; A: active atrial contraction; e': diastolic annular velocity. Participants with cardiac abnormalities excluded from the analysis.

Supplementary Table 2: Descriptive data for VBC participants stratified by urban or rural residence

		RURAL				URBAN		
		MALE		FEMALE		MALE		FEMALE
**Variable units**	**N**	**Mean SD/ Median IQR † / N %**	**N**	**Mean SD/ Median IQR † / N %**	**N**	**Mean SD/ Median IQR † / N %**	**N**	**Mean SD/ Median IQR † / N %**
**Clinical characteristics**								
Age (years)	460	**45.6** (1.06)	451	**45.9** (1.14)	364	**46.2** (1.06)	294	**46.3** (1.08)
Height (cm)	460	**166.8** (6.0)	451	**154.5** (5.8)	364	**167.6** (6.8)	294	**154.6** (5.7)
Weight (kg)	460	**66.0** (12.4)	451	**60.1** (12.9)	364	**71.7** (14.0)	294	**66.1** (12.6)
BMI (kg/m^2)	460	**23.6** (3.9)	451	**25.1** (5.1)	364	**25.4** (4.3)	294	**27.6** (4.8)
Body surface area (m^2)	460	**1.73** (0.17)	451	**1.57** (0.16)	364	**1.80** (0.18)	294	**1.64** (0.15)
Systolic blood pressure (mmHg)	460	**128.9** (18.8)	451	**123.0** (18.6)	364	**132.4** (19.6)	294	**128.0** (17.9)
Diastolic blood pressure	460	**79.7** (14.0)	451	**74.8** (11.4)	364	**79.6** (13.7)	294	**77.0** (10.9)
Pulse (bpm)	460	**79.1** (11.7)	451	**84.3** (11.1)	364	**82.8** (12.0)	294	**87.1** (11.2)
SLI score	364	**31.2** (5.6)	294	**30.7** (6.2)	460	**28.2** (6.9)	451	**27.5** (6.8)
Tobacco use								
Never		**277** (60.2)		**415** (92.0)		**222** (61.0)		**283** (96.3)
Ex-user		**36** (7.8)		**3** (0.7)		**33** (9.1)		**2** (0.7)
Current user		**147** (32.0)		**33** (7.3)		**109** (30.0)		**9** (3.1)
Alcohol consumption								
none		**173** (37.6)		**451** (100.0)		**148** (40.7)		**294** (100.0)
mild		**179** (38.9)		**0** (0.0)		**148** (40.7)		**0** (0.0)
moderate		**66** (14.4)		**0** (0.0)		**38** (10.4)		**0** (0.0)
heavy		**42** (9.1)		**0** (0.0)		**30** (8.2)		**0** (0.0)
**Cardiac size**								
LVIDd (mm)	460	**45.6** (4.57)	451	**42.9** (4.44)	364	**45.6** (4.55)	294	**43.2** (4.47)
LVIDs (mm)	460	**29.27** (3.12)	451	**27.39** (2.81)	364	**29.28** (3.19)	294	**27.53** (3.06)
Fractional shortening	460	**0.36** (0.04)	451	**0.36** (0.04)	364	**0.36** (0.03)	294	**0.36** (0.05)
PWT (mm)†	460	**9.0** (8.3, 9.3)	451	**8.7** (8.0, 9.0)	364	**9.0** (8.7, 9.7)	294	**9.0** (8.3, 9.0)
Septal thickness (mm)	460	**9.9** (1.1)	451	**9.3** (1.0)	364	**10.0** (1.2)	294	**9.4** (1.0)
Relative wall thickness †	460	**0.39** (0.36, 0.42)	451	**0.39** (0.36, 0.43)	364	**0.40** (0.37, 0.43)	294	**0.40** (0.37, 0.44)
LVEDV 4c (ml) †	460	**69.1** (59.8, 83.3)	451	**58.9** (49.2, 70.9)	364	**66.5** (55.6, 82.5)	294	**56.7** (49.6, 68.2)
LVESV 4c (ml) †	460	**23.7** (19.9, 28.4)	451	**20.2** (16.9, 23.9)	364	**23.0** (18.9, 28.3)	294	**19.7** (17.0, 23.3)
LV mass absolute (g) †	460	**117.0** (98.8, 134.0)	451	**97.3** (83.8, 112.0)	364	**116.0** (99.3, 136.0)	294	**102.0** (85.4, 118.0)
LV mass[i] indexed for BSA, (g/m^2)	460	**67.4** (58.9, 77.2)	451	**62.3** (54.9, 70.7)	364	**64.8** (57.3, 75.6)	294	**61.7** (54.9, 70.3)
**Categories of LV re-modelling/hypertrophy**								
None (n, %)		**330** (70.8)		**253** (68.4)		**296** (64.5)		**182** (60.9)
Concentric remodelling (n, %)		**124** (26.6)		**107** (28.9)		**141** (30.7)		**107** (35.8)
Concentric hypertrophy (n, %)		**3** (0.6)		**5** (1.4)		**8** (1.7)		**4** (1.3)
Eccentric hypertrophy (n, %)		**9** (1.9)		**5** (1.4)		**14** (3.1)		**6** (2.0)
**SYSTOLIC FUNCTION**								
LV ejection fraction (%) †	460	**65.4** (63.1, 67.4)	364	**65.4** (63.3, 67.2)	451	**65.8** (63.6, 67.6)	294	**65.5** (63.7, 67.2)
Low LV ejection fraction (n, %)		**3** (0.6)		**3** (0.8)		**1** (0.2)		**2** (0.7)
Average GLS	460	**-19.7** (2.0)	364	**-18.4** (2.2)	450	**-20.1** (2.1)	294	**-19.2** (2.3)

**Categories of GLS**								
Normal (n, %)		**431** (92.5)		**286** (77.3)		**423** (92.4)		**260** (87.0)
Borderline (n, %)		**27** (5.8)		**56** (15.1)		**26** (5.7)		**28** (9.4)
Abnormal (n, %)		**8** (1.7)		**28** (7.6)		**9** (2.0)		**11** (3.7)
**DIASTOLIC FUNCTION additionally excluded people in atrial fibrillation**								
Mitral - E (cm/sec) †	460	**74.8** (65.0, 85.4)	364	**73.9** (64.8, 82.8)	448	**77.6** (69.4, 89.3)	293	**80.4** (71.1, 89.6)
Mitral - A (cm/sec) †	460	**60.4** (53.0, 68.3)	364	**60.6** (53.2, 67.1)	448	**64.2** (56.1, 73.1)	293	**65.4** (58.6, 74.4)
Mitral E/A†	460	**1.2** (1.09, 1.43)	364	**1.24** (1.07, 1.39)	448	**1.24** (1.10, 1.39)	293	**1.22** (1.11, 1.35)
Deceleration time (ms)	460	**152.4** (31.0)	364	**142.0** (30.5)	448	**145.9** (31.6)	293	**138.8** (29.7)
Mitral E/average e' †	460	**7.8** (6.8, 9.1)	364	**8.1** (7.1, 9.4)	448	**8.5** (7.2, 9.5)	293	**8.8** (7.8, 10.0)
LA volume^i (ml/m^2) - 4c †	460	**11.8** (10.1, 13.9)	364	**11.3** (9.6, 13.3)	450	**12.1** (10.4, 14.4)	294	**11.7** (9.8, 13.7)
Average LA volume^i (ml/m^2)†	460	**11.6** (10.2, 13.3)	364	**11.3** (9.8, 13.0)	450	**12.2** (10.6, 14.2)	294	**11.7** (10.0, 13.3)
TR max m/sec	458	**174.7** (40.1)	364	**173.1** (37.6)	450	**180.1** (37.3)	294	**178.9** (38.7)
**Categories of LV diastolic dysfunction participants with normal LV ejection fraction only***								
None (n, %)		**449** (98.68)		**359** (99.45)		**447** (99.33)		**290** (98.98)
Indeterminate (n, %)		**6** (1.32)		**2** (0.55)		**3** (0.67)		**3** (1.02)
Dysfunction (n, %)		**0**		**0**		**0**		**0**
**Co-morbidities**								
Diabetes (n, %)		**113** (24.57)		**99** (27.20)		**79** (17.52)		**79** (26.87)
Hypertension (n, %)		**138** (30.00)		**130** (35.71)		**93** (20.62)		**80** (27.21)
Myocardial infarction (n, %)		**4** (0.87)		**4** (1.10)		**0** (0.00)		**0** (0.00)
Atrial fibrillation (n, %)		**0** (0.00)		**0** (0.00)		**1** (0.22)		**0** (0.00)
Overweight (BMI ≥25 kg/m^2), n (								
Obesity (BMI ≥30 kg/m^2), n (%)								

LV: left ventricle; LVIDD:LV internal diameter in diastole; LVISD: LV internal diameter in systole; PWT: Posterior wall thickness; GLS: Global longitudinal strain; E: passive ejection of left atrium; A: active atrial contraction; e': diastolic annular velocity. Participants with known cardiac disease excluded from the analysis.

Supplementary table 3. Comparison of Cardiac structure and function between IndEcho and other published studies form different ethnicities

	SABRE[1]			LOLIPOP[2]		NORRE study[3]	Sullere et al[4]	INDEA study[5]	IndEcho (Current study		WASE[6]	2015 ASE/EACVI guidelines
Ethnicity	European (n=427)	African Caribbean (n=143)	Migrant South Asians in UK (n=325)	European white (n=199)	Indian Asian migrants in the UK (n=259)	Caucasians (n=734)	Indians (n=707)	Indians (n=880)	NDBC (n=753)	VBC (n=1564)	Indians range	N=839
Age	70.1 (6.2)	70.9 (5.8)	69.2 (6.0)	51.8 (8.5)	50.6 (8.5)	45.8 (13.3)	40.7 (11.4)	39.7 (12.3)	46.13 (1.10)	45.93 (1.13)	-	45 (32 – 65)
Height, m	1.73 (0.07)	1.72 (0.06)	1.69 (0.06)	-	-	1.70 (0.96)	1.66 (1.01)	-	1.64 (0.09)	1.61 (0.09)	-	167 (160–174)
Weight, kg	80.4 (11.7)	71.7 (9.5)	79.3 (11.3)	-	-	69.5 (12.0)	74.6 (14.7)	-	76.4 (15.0)	65.6 (13.6)	-	67 (58–77)
BMI	-	-	-	24.4 (2.7)	25.0 (2.6)	24.0 (3.0)	27.2 (4.9)	23.7 (2.1)	28.4 (5.0)	25.2 (4.7)	-	-
BSA				1.90 (0.2)	1.80 (0.2)	1.80 (0.2)		1.69 (0.15)	1.83 (0.20)	1.69 (0.19)	-	
LVIDd, cm	4.52 (0.02)	4.46 (0.03)	4.39 (0.02)	-	-	4.43 (0.48)	4.62 (0.44)	4.22 (0.39)	4.82 (0.61)	4.40 (0.52)	3.4 to 5.2 (M)<br>3.2 to 4.9 (M)	4.2 to 5.8 (M)<br>3.8 to 5.2 (F)
LVEDV, ml	84.6 (0.9)	79.0 (1.6)	77.3 (1.02)			92.8 (24.8)	88.1 (20.7)	71.5 (17.5)	55.3 (14.7)	63.1 (15.2)	47 to 113 (M)<br>40 to 91 (F)	62 to 150 (M)<br>46 to 106 (F)
IVS, cm	1.15 (0.1)	1.25 (0.02)	1.10 (0.1)	-	-	0.86 (0.2)	1.07 (0.11)	0.89 (0.1)	0.96 (0.2)	0.97 (0.1)	-	-
PWT, cm	1.02 (0.01)	1.09 (0.01)	1.00 (0.01)	-	-	0.88 (0.2)	1.06 (0.1)	-	0.87 (0.2)	0.88 (0.1)	-	-
RWT	0.46 (0.004)	0.49 (0.007)	0.46 (0.004)	0.35 (0.07)	0.37 (0.07)	-	0.47 (0.05)	-	0.38 (0.08)	0.40 (0.06)	-	-
LV mass, g	176.3 (2.1)	190.8 (3.7)	159.8 (2.4)	159.3 (45.3)	132.5 (33.4)	126.8 (37.4)	176.8 (37.7)	-	126.2 (33.5)	110.5 (27.6)	-	-
LV mass/BSA, g/m2	92.9 (1.04)	101.0 (1.8)	90.2 (1.2)	92.1 (2.5)	80.2 (1.9)	69.9 (17.5)	96.9 (22.2)	-	69.6 (18.3)	65.4 (13.8)	40 to 88 (M)<br>37 to 78 (M)	43 – 102 (M)<br>36 – 89 (F)
E/A ratio	-	-	-	1.2 (0.3)	1.2 (0.4)	-	1.4 (0.4)	1.5 (0.5)	1.1 (0.23)	1.2 (0.27)	-	-
EF (%)	-	-	-	62 (5)	62 (6)	63.9 (4.9)	61.2 (5.1)	61.7 (5.9)	64.0 (4.4)	65.3 (3.0)	58 to 69 (M)<br>59 to 69 (F)	52 – 72 (M)<br>54 – 74 (F)
GLS%	-	-	-	-	-	-	-	– 21.0 (2.9)	-18.14 (3.14)	-19.4 (2.2)	-17 to -25 (M)<br>-18 to -26 (F)	-

1.Park et al., SABRE study. Hypertension. 2013; 2. Chahal et al., LOLIPOP study. Heart 2010; 3. Kou et al., The NORRE study. *European Heart Journal–Cardiovascular Imaging* 2014; 4. Sullere et al. Indian Heart Journal 2018; 5. Sengupta et al., INDEA study *Int J Cardiovasc Imaging.* 2021; 6. Asch et al. WASE study *J Am Soc Echocardiogr.* 2019; 7. Lang et al.,ASE/EACVI guidelines. *J Am Soc Echocardiogr.* 2015
